# LC3-associated phagocytosis in macrophage responses to *Paracoccidioides* spp.

**DOI:** 10.1590/0074-02760250089

**Published:** 2026-03-06

**Authors:** Getúlio Pereira de Oliveira, Herdson Renney de Sousa, Kaio César de Melo Gorgonha, Lara Laís Montalvão Tomaz, Tatiana Karla dos Santos Borges, Kellyanne Teixeira Rangel, Scott Fabricant, Fernanda Koser Gustafson, Lucas Friaça Albuquerque, Angelo Rossi, Fabián Andrés Hurtado, Hugo Costa Paes, Arturo Casadevall, Ildinete Silva-Pereira, Maria Sueli Soares Felipe, Patrícia Albuquerque, André Moraes Nicola

**Affiliations:** 1 Northeastern University, Barnett Institute for Chemical & Biological Analysis, Boston, MA, USA; 2 Universidade Católica de Brasília, Programa de Pós-Graduação em Ciências Genômicas e Biotecnologia, Brasília, DF, Brasil; 3 Universidade de Brasília, Faculdade de Medicina, Brasília, DF, Brasil; 4 Universidade Católica de Brasília, Escola de Medicina, Brasília, DF, Brasil; 5 Universidade de Brasília, Instituto de Ciências Biológicas, Brasília, DF, Brasil; 6 Johns Hopkins University, Johns Hopkins Bloomberg School of Public Health, Baltimore, MD, USA; 7 Universidade de Brasília, Faculdade de Ciências e Tecnologias da Saúde, Brasília, DF, Brasil; 8 Cornell University, Weill Cornell Medicine, Jill Roberts Institute for Research in Inflammatory Bowel Disease, New York, NY, USA; 9 Rockefeller University, Laboratory of Molecular Immunology, New York, NY, USA; 10 Universidade de São Paulo, Instituto Nacional de Ciência e Tecnologia Fungos Patogênicos Humanos, São Paulo, SP, Brasil

**Keywords:** autophagy, LC3-associated phagocytosis (LAP), macrophage, Paracoccidioides, fluorescence microscopy

## Abstract

**BACKGROUND:**

Paracoccidioidomycosis (PCM) is a systemic infection that is endemic to Latin America, caused by thermodimorphic fungi from the *Paracoccidioides* genus. These fungi are facultative intracellular parasites of macrophages. LC3-associated phagocytosis (LAP), a non-canonical form of autophagy, plays a critical role in the response of these phagocytes to similar pathogens.

**OBJECTIVES:**

In this study, we investigated the role of LAP in the macrophage responses to *Paracoccidioides* spp.

**METHODS:**

We detected LAP in macrophages infected with *Paracoccidioides* spp by immunofluorescence microscopy with antibodies to LC3. Piceatannol and diphenyleneiodonium chloride (DPI), respectively Syk and nicotinamide adenine dinucleotide phosphate oxidase (NADPH) inhibitors, were used to understand the role their pathways played. To determine the function of LAP, we targeted *ATG5*, a key autophagy gene, by RNA interference.

**FINDINGS:**

We observed LC3 recruitment to phagosomes containing *Paracoccidioides* spp. in RAW264.7 and J774.16 cell lines and in bone marrow-derived macrophages. *ATG5* RNA interference reduced the antifungal activity of J774.16 cells, highlighting the importance of LC3 recruitment for effective fungal control. Interestingly, pharmacological inhibition of Syk kinase and NADPH oxidase pathways, essential for LAP against *Aspergillus fumigatus* and *Candida albicans*, did not impair LAP against *P. brasiliensis*.

**MAIN CONCLUSIONS:**

This suggests distinct triggering mechanisms, possibly due to differences in the fungal cell surface composition. These findings suggest that LAP plays a significant role in the host defense against *Paracoccidioides* spp. and may represent a promising target for host-directed PCM therapies.

## INTRODUCTION

The *Paracoccidioides* genus includes thermodimorphic fungi that can be isolated from the soil,^1^ especially around armadillo burrows.^2^ Upon inhalation by humans, they can cause paracoccidioidomycosis (PCM) ― one of the most prevalent systemic mycoses in Latin America.^3^ PCM manifests as either chronic disease localized to mucosae and lungs or acute systemic infection, with high fungal burden in lymph nodes and the spleen.^4^ The disease poses significant public health burden, with annual incidence as high as 9.4 per 100,000 and a case-fatality rate of approximately 6% in endemic regions.^5^

Macrophages play a central role in the immune response to *Paracoccidioides* spp., with their functional states (pro-inflammatory M1 versus anti-inflammatory M2) influencing disease outcomes.^6^ Activated M1 macrophages secrete cytokines such as IL-12 and produce nitric oxide, which are critical for fungal clearance.^7^ However, *Paracoccidioides* infections are sometimes associated with immune dysregulation, leading to granulomatous inflammation and impaired fungal control that result in active disease.^8^

Current PCM treatments, including sulfonamides, amphotericin B, and azole derivatives, are lengthy, costly, and often associated with adverse effects or relapse.^9^ This underscores the need for novel therapeutic approaches, including strategies targeting host immune mechanisms.^10^

Autophagy, a conserved eukaryote homeostatic process,^11^ ^12^ ^13^ plays a key role in host immunity against intracellular pathogens including fungi.^14^ ^15^ Among its variants, LC3 associated phagocytosis (LAP) is triggered during macrophage responses to pathogens such as *Aspergillus fumigatus*,^16^ ^17^ *Cryptococcus neoformans*,^18^ ^19^ *Candida albicans*,^18^ ^20^ ^21^ *Histoplasma capsulatum*,^22^ ^23^ *Talaromyces marneffei*,^24^ ^25^ and *Saccharomyces cerevisiae*-derived zymosan.^26^ Given its importance in other fungal infections, understanding LAP’s role in the immune response to *Paracoccidioides* spp. could reveal novel therapeutic targets.

In this study, we investigated whether macrophages use LAP to respond to *Paracoccidioides* spp. and explored the signaling pathways involved. Our findings provide insights into the mechanisms underpinning macrophage antifungal responses and highlight LAP as a potential target for host-directed therapies.

## MATERIALS AND METHODS

**Fungal and cell cultures** - The Pb18 and Pb01 isolates of *P. brasiliensis* and *P. lutzii*, respectively, were maintained in Fava-Netto’s medium (1% w/v peptone, 0.5% w/v yeast extract 0.3% w/v proteose peptone, 0.5% w/v beef extract, 0.5% w/v NaCl, 4% w/v glucose, and 1.4% w/v agar, pH 7.2), at 37ºC. Cultures no older than five days from the last passage were used for experiments. For fungicidal activity experiments, fungal colony forming units (CFUs) were counted by plating in brain-heart infusion (BHI) agar supplemented with horse serum and *P. brasiliensis* strain 192 conditioned medium. All these culture conditions are conducive to the yeast phenotype, which we used for all experiments.

**Cell lines** - The mouse macrophage cell lines RAW 264.7, and J774.16 were used for the detection of LAP in vitro. HEK 293T, and J774.16 were used for transfection and transduction assays, respectively. Cells were kept in 100-mm Petri dishes with supplemented Dulbecco’s Modified Eagle’s Medium (DMEM) with 1% non-essential amino-acid solution and 10% of fetal bovine serum (FBS) and incubated at 37ºC and 5% CO2.

**Animals and primary cells** - C57BL/6 mice were bred at the Animal Center of the University of Brasília Institute of Biological Sciences with food and water ad libitum. All procedures were performed in accordance with national and institutional guidelines for animal care and were approved by the university’s Institutional Animal Care Use Committee (Proc. UnB Doc 52657/2011). Bone marrow-derived macrophages were generated from bone marrow cells from six- to 12-week old C57BL/6 mice as previously described,^27^ a condition that results in cultures with approximately 80% macrophages.^28^ Briefly, 2 x 106 bone marrow cells were plated on non-tissue treated 100-mm Petri dishes with RPMI 1640 supplemented with 10% heat-inactivated FBS (Thermo Fisher), 50 µg/mL gentamicin, 50 µM 2-mercaptoethanol (Sigma-Aldrich) and 20 ng/mL recombinant GM-CSF (Peprotech). The cultures were incubated for eight days at 37ºC in a humidified 5% CO2 atmosphere. On day 3, 10 mL of fresh complete RPMI was added to the culture. Half of the medium was removed on day 6 and new complete RPMI was added. Attached bone marrow derived macrophages (BMMs) were collected on day 8 with TrypLE™ Express (Thermo Fisher). Cell viability was consistently > 95%, as measured by trypan blue exclusion.

**Production of ATG5 shRNA lentiviral vectors** - For the transfection assay we used the third generation lentiviral packaging vectors pRSV-Rev, pMDLg/pRRE and pMD2.G. The pLK0.1 lentiviral vector was used to transduce shRNAs targeting murine *ATG5*. As negative control, we used a similar vector encoding an shRNA that targets the enhanced green fluorescent protein (EGFP) gene, a sequence that is not present in the cell. Plasmid expansion was performed in competent *Escherichia coli* (Omnimax T1, Thermo Fisher) in lysogeny broth (LB) with 100 µg/mL ampicillin. Plasmids were purified with the GenElute Plasmid DNA Miniprep Kit (Sigma-Aldrich), quantified using the Qubit fluorometer (Thermo Fisher) and stored at -20ºC.

HEK 293T cells were trypsinized and harvested at 90-95% confluence. Cells were reseeded at a concentration of 3.75 x 105 per mL onto a six-well plate containing 2 mL of supplemented DMEM per well. A mix containing OptiMEM media, Lipofectamine 2000 (Invitrogen), and the assembly media containing the packing plasmids plus the pLKO.1 vectors encoding each shRNA were added to each well. After 6 h of incubation, 2 mL of supplemented DMEM were added to the cells, and the supernatant was collected after 12 and 24 h. Supernatants were centrifuged at 200 x g for 5 min to remove dead cells and debris, and the resulting supernatant was centrifuged again at 20.000 x g for 90 min at 4ºC. Pellets containing lentivirus were resuspended and stored at -80ºC.

**J774.16 cell transduction with ATG5 shRNA lentivirus** - After J774.16 cells reached 95% confluence, they were harvested by trypsinization and counted. They were reseeded onto a 96-well plate containing supplemented DMEM, at 104 cells/well. In the following day, the cell culture medium was exchanged for supplemented DMEM with 8 µg/mL hexadimethrine bromide (Polybrene), and 5, 50 or 100 µL of lentivirus harboring each shRNA were added to the cells. In the next day, the medium was exchanged for fresh supplemented DMEM, and after one more day, transduced cells were selected with puromycin at 0.5 µg/mL (Thermo Fisher). After 48 h of selection, untransduced dead cells were removed from the supernatant. For the second round of selection, these transduced J774.16 cells were harvested and seeded onto a six-well plate with supplemented DMEM containing puromycin at 5 µg/mL. After reaching confluence, the cells were harvested, and total RNA was extracted using Trizol® (Thermo Fisher). RNA was analyzed by electrophoresis with 1% agarose, gene expression evaluated by quantitative polymerase chain reaction (qPCR) using SYBR® Green to generate the amplification signal, and the fold-changes were calculated using the 2-ΔΔCt method.

**Fungal killing assay** - For CFU experiments, stably transduced J774.16 cells were seeded onto a 96-well plate at 2 x 104 cells per well in supplemented DMEM and activated with murine interferon (IFN)-γ at 200 U/mL plus LPS at 1 µg/mL. After 24 h of activation and adhesion, the J774.16 cells were co-incubated with *P. brasiliensis* suspensions. To prepare these fungal suspensions, *P. brasiliensis* yeast cells were scraped from solid media and suspended in phosphate buffered saline solution (PBS). After vortexing with 2 - 4 mm glass beads for 30 s, large clumps were removed by decanting and the suspension strained through a 40 µm cell strainer. The viable cell density on the resulting suspension was counted in a hemocytometer using the vital dye Phloxine B. J774.16 cells were co-incubated with *P. brasiliensis* for 24 h, with a multiplicity of infection (MOI) of one. After this period, macrophages were lysed with sterile distilled water and fungal CFUs were counted by plating the same dilution for each well onto BHI agar plates and incubating at 37ºC until colonies appeared (five to seven days). Controls included untransduced J774.16 cells and wells with no macrophages. The experiment was repeated independently three times in different days, each with four or five wells per condition.

**Co-incubation of macrophages and Paracoccidioides spp. for LC3 immunofluorescence** - In different experiments, macrophages were either plated onto glass-bottom dishes (Mattek®) or on 24-well plates with sterile circular coverslips for 24 h. *P. brasiliensis* yeast cells were harvested from five-day old culture plates by scraping the surface of the fungal mat, vortexing the cells in PBS, passing the suspension through a 40 µm cell strainer and measuring cell density in a hemocytometer. Fungal cells were inoculated onto the plated macrophages at a MOI of one. The dishes were incubated for 12 to 24 h at 37ºC in the presence of 5% CO2 to allow infection. Afterwards, the plates were processed for immunofluorescence as described below. In some experiments, we added the Syk-selective tyrosine kinase inhibitor piceatannol (PIC) at 30 µM (Invivogen, San Diego, CA, catalog # tlrl-pct) or the nicotinamide adenine dinucleotide phosphate oxidase (NADPH) inhibitor diphenyleneiodonium chloride (DPI) at 20 µM (Sigma-Aldrich, Sant Louis, Missouri, catalog #D2926) to the dishes. Inhibitors were added 10 min before stimulation and remained in culture for the duration of the experiments. To minimize potential bias in the quantification of LC3 recruitment, all scoring was performed by a trained technician blinded to the experimental groups. Additionally, *C. albicans* infection was included as a positive control for LAP induction, confirming the reliability of the scoring methodology.

**Fig. 1:**
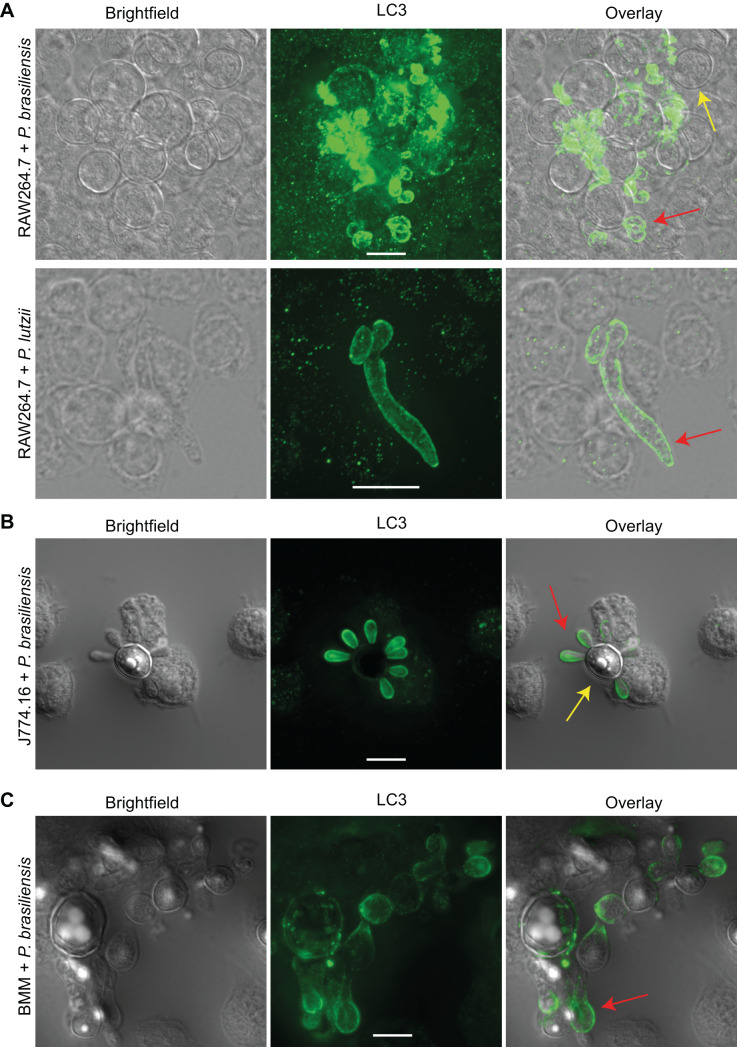
LC3 associated phagocytosis (LAP) is activated in murine macrophages against *Paracoccidioides* spp. (A) LAP was detected after 24 h in RAW264.7 incubated with *Paracoccidioides brasiliensis* and *P. lutzii*. (B) The same phenomenon was confirmed after 12 h of the *P. brasiliensis* interaction with J774.16 and (C) bone marrow derived macrophages (BMM). Experiments were repeated at least twice on different days and had similar results. The red arrows indicate fungi that are surrounded by LC3, whereas the yellow arrows point to fungi that are not. Scale bars: 10 µm.

**LC3 immunolocalization** - After 12 or 24 h of infection, the cells were fixed with ice-cold methanol for 10 min and washed with PBS. After that, they were incubated with a 1% BSA solution in PBS containing primary antibody (rabbit polyclonal IgG against human LC3, 1:1000 dilution, Santa Cruz Biotechnology) for 1 h at 37ºC. They were then washed three times with PBS and incubated with the secondary antibody (goat IgG against rabbit IgG conjugated with AlexaFluor® 488, Thermo Fisher Scientific) diluted 1:2000 in the same conditions as the primary one. Calcofluor White at 1 g/L was used in some experiments to label the fungal cell wall. After straining, the cells were washed three times with PBS and the glass-bottom dishes (or coverslips) were mounted with ProLong Gold Antifade Mountant (Thermo Fisher Scientific). We included two negative controls [Supplementary data (Fig. 1A)]: (1) unstained macrophages and *Paracoccidioides* cells (autofluorescence); (2) co-cultures of macrophage and *Paracoccidioides* cells with the secondary but without the primary antibody (non-specific binding of the secondary antibody). Additional experiments with no macrophages, only *Paracoccidioides* spp. cells, showed that the LC3 antibody does not bind to the fungal cell [Supplementary data (Fig. 1B)]. Samples were documented in a Zeiss Axio Observer Z1 epifluorescence microscope equipped with a 63x NA 1.4 oil immersion objective and a cooled CCD camera. Image stacks were deconvolved with a constrained iterative algorithm on Zeiss ZEN and then processed on ImageJ and Adobe Photoshop. No non-linear modifications were made to the images.

**Statistical analysis** - For *ATG5* knockdown, analysis of variance (ANOVA) and Dunnett’s multiple comparison pos-hoc tests were performed on Graphpad Prism. For LAP quantitative analysis, a Fisher’s exact test was performed to compare proportions of fungi on LC3-positive vacuoles on Graphpad Prism. For CFU analysis, a mixed-analysis ANOVA was used, with shRNA as a fixed effect and replicate as a random factor. Pairwise comparisons were made using Tukey’s HSD to correct for multiple comparisons. This CFU analysis was performed in R using the MultComp package.^29^

## RESULTS

**Fig. 2:**
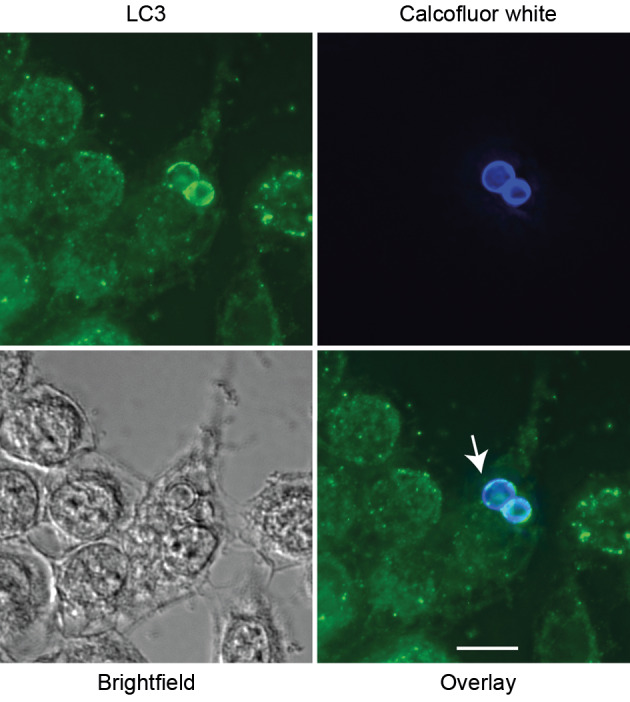
accumulation and colocalization of LC3 in the *Paracoccidioides* cell wall. *Paracoccidioides brasiliensis* cells were dyed with calcofluor white before their interaction with J774.16 macrophages. LC3 associated phagocytosis (LAP) was detected after 24 h of interaction, and the signal could be observed around the fungal cell wall (arrow). Scale bar: 10 µm.

**LC3 is recruited to phagosomes containing Paracoccidioides spp. in murine macrophages** - To investigate whether macrophages use LAP against *Paracoccidioides* spp., we performed immunofluorescence microscopy. LC3 was detected in phagosomes containing either *P. brasiliensis* or *P. lutzii* across multiple macrophage types (RAW264.7, J774.16, and BMM) after 12 or 24 h of co-incubation (Fig. 1A-C). Controls for autofluorescence and non-specific secondary antibody binding were negative [Supplementary data (Fig. 1)]. Using calcofluor white, we could see that LC3 accumulates around the fungal cell wall (Fig. 2).

Interestingly, LC3 localization was more frequently observed around fungal daughter cells compared to mother cells (Fig. 1B). Additionally, we detected LC3 around *Paracoccidioides* spp. cells that were clearly outside macrophages. A final striking observation is that LC3 recruitment was not universal, as only a subset of phagosomes displayed LC3. These findings demonstrate that LAP is activated in macrophages during interactions with *Paracoccidioides* spp., albeit in a selective manner.

**Fig. 3:**
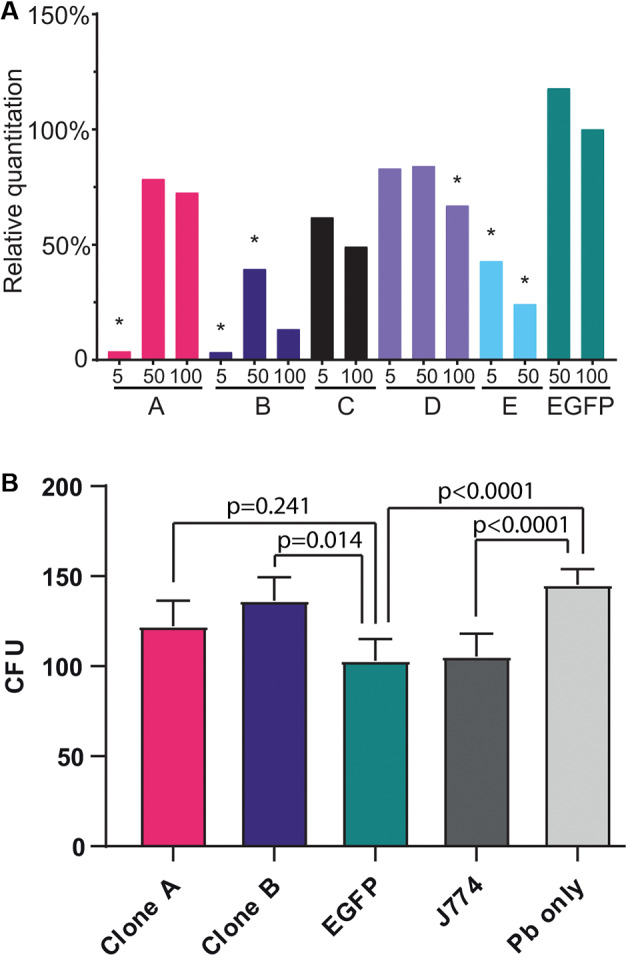
*ATG5* knockdown efficiency and impact on fungal burden in *Paracoccidioides*-infected macrophages. (A) Relative quantification of *ATG5* expression in J774.16 macrophages transduced with five different lentiviral shRNA constructs that target *ATG5* (clones A-E) compared to a negative control vector that targets a gene that is not present on the cells (EGFP). Bars represent *ATG5* mRNA levels at three different multiplicities of infection (MOI: 5, 50, 100). Clones A and B showed the strongest knockdown efficiency (asterisks indicate p < 0.05 compared to the EGFP negative control). (B) Fungal burden [colony forming unit (CFU) counts] in *Paracoccidioides brasiliensis* (Pb)-infected macrophages at 48 h post-infection. *ATG5*-silenced clones A and B are compared to negative controls including untransduced macrophages (J774) and cells in which the shRNA target is not present (EGFP). “Pb only” indicates fungal growth in the absence of macrophages. Data represent mean ± standard deviation (SD) from three independent experiments. Statistical analysis was performed using one-way analysis of variance (ANOVA) with Tukey’s HSD multiple comparison post-hoc test; p-values are shown above the bars.

**LAP is important in the murine macrophage response to P. brasiliensis** - To explore the functional significance of LC3 recruitment, we conducted loss-of-function experiments targeting *ATG5*, a gene essential for autophagy and LAP.^26^ J774.16 macrophages were transduced with lentiviral vectors encoding shRNAs against *ATG5*. Two shRNAs (clones A and B) achieved significant knockdown, reducing *ATG5* expression by approximately 97% compared to controls (Fig. 3A).

A significant reduction in antifungal activity in comparison with the non-silenced control was observed in *ATG5*-silenced clone B, but not on clone A. Targeting a sequence that does not exist on the macrophage, EGFP, had no effect, confirming that the reduced antifungal activity is specific for *ATG5* silencing in clone B (Fig. 3B). These findings indicate that LAP contributes to macrophage antifungal responses against *Paracoccidioides* spp.

**Fig. 4:**
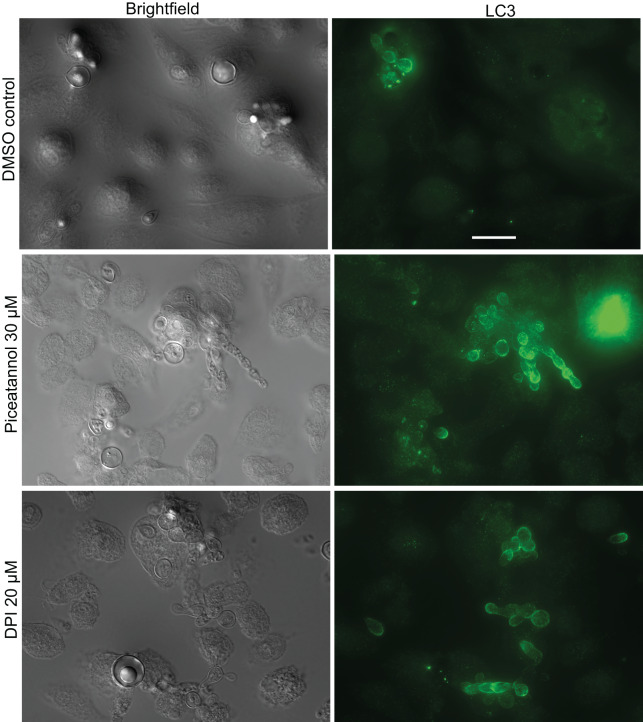
effect of Syk and nicotinamide adenine dinucleotide phosphate oxidase (NADPH) inhibitor on bone marrow derived macrophages (BMM) LC3 associated phagocytosis (LAP) against *Paracoccidioides brasiliensis*. Piceatannol (PIC) at 30 µM) and diphenyleneiodonium chloride (DPI) at 20 µM were used to inhibit Syk and NADPH oxidase, respectively. Syk inhibition led to an increase in LAP, whereas NADPH oxidase inhibition did not seem to affect LAP. White arrows indicate the dotted pattern of LC3 protein inside macrophages (not associated with phagocytosed fungi). Scale bar: 20 μm.

**Distinct mechanisms of LAP activation** - To determine whether LAP activation against *Paracoccidioides* spp. involves similar pathways to those used against other fungi, we used pharmacological inhibitors targeting Syk kinase and the NADPH oxidase, which are required for LAP induction in macrophages infected with *C. albicans* ^21^ and *A. fumigatus*.^17^ Syk inhibition by piceatannol increased LC3 recruitment to phagosomes containing *P. brasiliensis*, while NADPH oxidase inhibition with DPI had no effect (Fig. 4 and Table).

**TABLE:** Recruitment of LC3 to vacuoles containing *Paracoccidioides brasiliensis* and *Candida albicans* in primary macrophages. Bone marrow derived macrophages (BMMs) were infected with either fungi in the presence or absence of Syk and nicotinamide adenine dinucleotide phosphate oxidase (NADPH) inhibitors and processed for immunofluorescence microscopy as shown in Fig. 4. Individual macrophages with at least one phagocytosed yeast were then observed and scored based on the presence or absence of LC3 surrounding the internalized fungi

**Fungus**	**Treatment**	**Macrophages^a^**	**LAP^b^**	**Percentage^c^**	**Odds ratio**	***p-value* ^d^**
*P. brasiliensis*	DMSO	218	39	17.9%	-	-
	30 µM PIC	208	62	29.8%	1.666	0.0267
	20 µM DPI	222	47	21.2%	1.183	0.4822
*C. albicans*	DMSO	103	58	56.3%	-	-
	30 µM PIC	75	30	40.0%	0.71	0.2314
	20 µM DPI	119	25	21.0%	0.373	0.0003

a: number of macrophages that were observed to have phagocytosed at least one *P. brasiliensis* cell; b: number of macrophages among those in which the phagocytosed fungi were surrounded by LC3; c: percentage of macrophages in which the phagocytosed fungi were surrounded by LC3; d: Fisher’s exact test; LAP: LC3 associated phagocytosis; DMSO: dimethyl sulfoxide; PIC: piceatannol; DPI: diphenyleneiodonium chloride.

As these results differ from what was observed with other fungi, we also repeated the experiments with *C. albicans*. As shown on Supplementary data (Fig. 2) and Table, the replication of previous studies^21^ confirms our results.

## DISCUSSION

Our findings demonstrate that macrophages deploy LAP as part of their response to *Paracoccidioides* spp. Using immunofluorescence, we observed LC3 recruitment to phagosomes containing different *Paracoccidioides* spp. across multiple macrophage types, including RAW264.7, J774.16, and bone marrow-derived macrophages. Interestingly, this recruitment was selective, occurring in only a subset of phagosomes, with LC3 frequently observed around fungal daughter cells but not mother cells. This pattern may reflect differences in the composition of the fungal cell wall, as surface microbial molecular patterns are key LAP triggers.^22^ Additionally, LC3 was detected around apparently extracellular fungal cells, a phenomenon that had been previously observed with *C. neoformans*.^18^ This could represent non-lytic exocytosis (vomocytosis) of the phagosomal contents, a process previously reported in interactions between *Paracoccidioides* spp. and amoebae;^30^ testing this speculation, however, is beyond the scope of the present work.

The functional significance of this LC3 recruitment was confirmed through loss-of-function experiments targeting *ATG5*, a gene that is essential for autophagy and LAP. *ATG5* knockdown significantly reduced macrophage antifungal activity, as evidenced by increased fungal viability in CFU assays. These results support the role of LAP in macrophage-mediated control of *P. brasiliensis*. However, the modest reduction in antifungal activity observed, even in *ATG5*-silenced macrophages, suggests that LAP operates as part of a broader immune response rather than acting as the sole antifungal process.

The CFU results align with previous work from our group on macrophages and dendritic cells from susceptible and resistant mouse strains,^31^ an indication that LAP could play a role in in vivo responses to *Paracoccidioides* spp. However, care is warranted in reaching strong conclusions in this regard because on macrophages infected with the closely related *H. capsulatum*, LAP is actually detrimental to the host and exploited by the fungus to survive.^23^ The literature on antifungal LAP in macrophages is rife with apparent contradictions that highlight how complex this mechanism is. In macrophages infected with *C. neoformans* in vitro, for instance, we found that LAP was host-protective^18^ but another group found it benefited the pathogen.^19^ ^32^ In invasive candidiasis models, we^18^ and others^17^ ^33^ ^34^ found that autophagy was host-protective, whereas other experiments concluded it was not necessary for proper responses to *C. albicans*.^35^

Mechanistically, our results suggest distinct pathways of LAP activation in macrophages infected with *Paracoccidioides* spp. compared to other fungi such as *C. albicans*,^36^ *A. fumigatus* ^17^ or *Histoplasma capsulatum*.^37^ Pharmacological inhibition of Syk kinase, which is essential for LAP activation in *C. albicans*, unexpectedly increased LC3 recruitment to phagosomes containing *P. brasiliensis*. Similarly, the inhibition of NADPH oxidase, another critical pathway for LAP activation in other fungal infections, had no effect on LAP in *P. brasiliensis*. The divergent effects of these inhibitors suggest that macrophages rely on distinct signaling pathways to activate LAP in response to *Paracoccidioides* spp. Differences in fungal cell wall composition, particularly in exposure of α- and β-glucans and other pattern recognition receptor ligands,^38^ ^39^ may underlie these mechanistic differences.

Despite the strength of these findings, several limitations should be considered. The in vitro nature of our experiments restricts their applicability to the complex immune environment of in vivo infections. Additionally, the limited antifungal activity observed in macrophage cultures may reflect intrinsic limitations of the J774.16 cell line. Future studies should investigate LAP’s role in murine PCM models, including its interaction with other immune pathways, such as cytokine signaling and adaptive immune responses.

Nevertheless, this activation of LAP against *Paracoccidioides* spp. has potential therapeutic implications. Targeting host-directed pathways with pharmacological activators of LAP or compounds that enhance downstream effector functions to enhance macrophage antifungal activity could be explored as adjunctive PCM therapies. This would reduce reliance on current antifungal therapies, which are often associated with toxicity, long treatment, and the risk of relapse;^40^ ^41^ ^42^ ^43^ and possibly lead to better outcomes. A new generation of specific autophagy-modulating compounds^35^ ^44^ ^45^ ^46^ may hold promise in this regard.

In summary, our findings add to the growing body of scientific evidence that the intracellular events after the uptake of fungal cells by phagocytes are protean and more research is needed to generate a consistent picture of these phenomena. Nevertheless, they provide new insights into antifungal immunity and underscore the potential of LAP as a target for host-directed therapies to combat systemic mycoses such as PCM.

## SUPPLEMENTARY MATERIALS

Supplementary material

## Data Availability

The contents underlying the research text are included in the manuscript.
