# Supplementary material for: LC3-associated phagocytosis in macrophage responses to Paracoccidioides spp
Source: Mem Inst Oswaldo Cruz. 2026 Mar 6;121:e250089. doi: 10.1590/0074-02760250089 (PMC12965332; doi:10.1590/0074-02760250089)
Supplement: Supplementary material [file 1678-8060-mioc-121-e250089-s1.pdf]

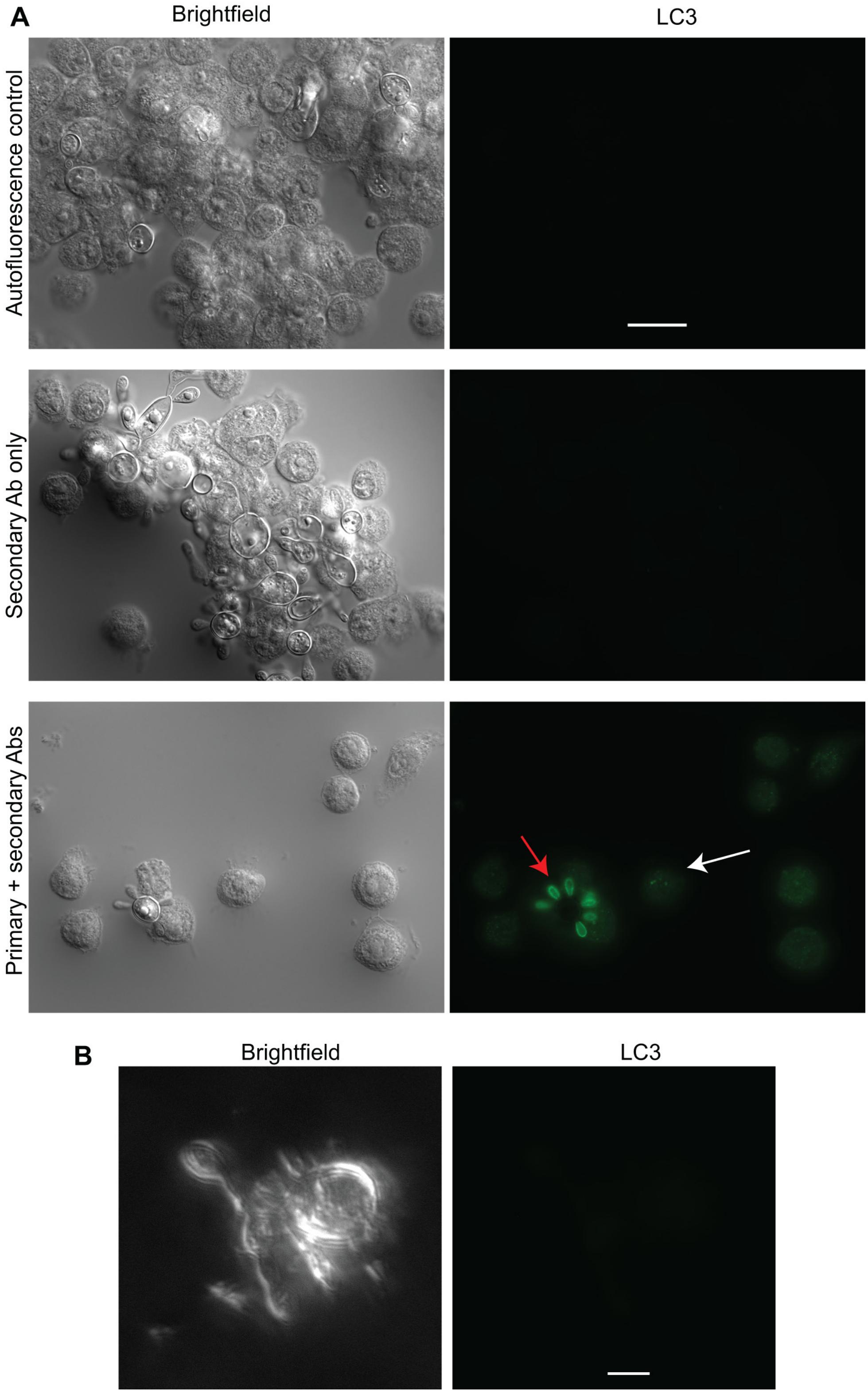

Fig. 1: immunofluorescence microscopy controls. (A) Autofluorescence and non-specific secondary antibody binding controls were used to verify our LC3-associated phagocytosis (LAP) experiments. No fluorescence was identified in either control experiment. For comparison, the bottom panel shows a representative image from a sample that was fully processed for LAP. This image is the whole field from which Fig. 1, panel B was made, and was collected using the same exposure time as the controls on the first two panels. The white arrow indicates a macrophage that has not phagocytosed *Paracoccidioides* spp., with the expected pattern of LC3 distribution - diffuse cytoplasmic staining accompanied by bright spots (autophagosomes). The red arrow indicates phagocytosed fungi that are LAP-positive. Scale bar: 10 µm. (B) Additional negative control, testing if the antibodies we used recognize fungal LC3 homologs. *Paracoccidioides* spp. cells were prepared with the same protocol as a full experiment, and the cells imaged afterwards with an exposure time that is similar to the one used for panel A. Scale bar: 5 µm.

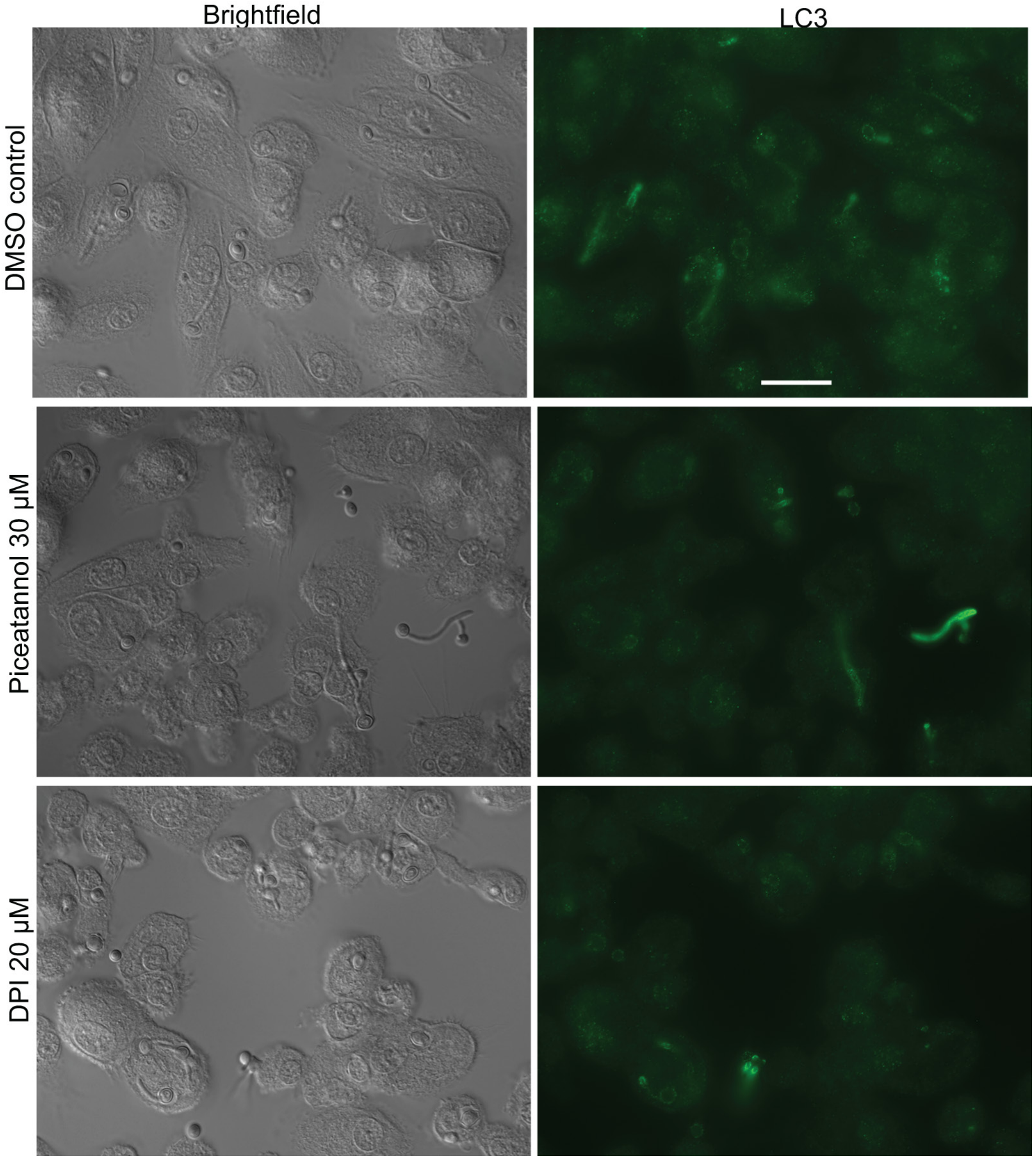

Fig. 2: inhibition of Syk and nicotinamide adenine dinucleotide phosphate oxidase (NADPH) in macrophages infected with *Candida albicans*. BMMs were infected with *C. albicans* for 12 h in the presence of Syk and NADPH oxidase inhibitors. The cells were then used for LC3 immunofluorescence, showing a decrease in the proportion of macrophages that deployed LC3-associated phagocytosis (LAP) against the internalized fungus. Scale bar: 20 µm.
